# A trivariate meta-analysis of diagnostic studies accounting for prevalence and non-evaluable subjects: re-evaluation of the meta-analysis of coronary CT angiography studies

**DOI:** 10.1186/1471-2288-14-128

**Published:** 2014-12-04

**Authors:** Xiaoye Ma, Muhammad Fareed K Suri, Haitao Chu

**Affiliations:** Division of Biostatistics, School of Public Health, University of Minnesota, A460 Mayo Building, MMC 303, 420 Delaware St. SE, 55455 Minneapolis, MN USA; Department of Neurology, University of Minnesota, MMC 295, 420 Delaware St. SE, 55455 Minneapolis, MN USA

**Keywords:** Meta-analysis, Diagnostic test, Non-evaluable subjects

## Abstract

**Background:**

A recent paper proposed an intent-to-diagnose approach to handle non-evaluable index test results and discussed several alternative approaches, with an application to the meta-analysis of coronary CT angiography diagnostic accuracy studies. However, no simulation studies have been conducted to test the performance of the methods.

**Methods:**

We propose an extended trivariate generalized linear mixed model (TGLMM) to handle non-evaluable index test results. The performance of the intent-to-diagnose approach, the alternative approaches and the extended TGLMM approach is examined by extensive simulation studies. The meta-analysis of coronary CT angiography diagnostic accuracy studies is re-evaluated by the extended TGLMM.

**Results:**

Simulation studies showed that the intent-to-diagnose approach under-estimate sensitivity and specificity. Under the missing at random (MAR) assumption, the TGLMM gives nearly unbiased estimates of test accuracy indices and disease prevalence. After applying the TGLMM approach to re-evaluate the coronary CT angiography meta-analysis, overall median sensitivity is 0.98 (0.967, 0.993), specificity is 0.875 (0.827, 0.923) and disease prevalence is 0.478 (0.379, 0.577).

**Conclusions:**

Under MAR assumption, the intent-to-diagnose approach under-estimate both sensitivity and specificity, while the extended TGLMM gives nearly unbiased estimates of sensitivity, specificity and prevalence. We recommend the extended TGLMM to handle non-evaluable index test subjects.

## Background

In studies of meta-analysis of diagnostic test comparing an index test with a reference test, non-evaluable test outcome is an important issue that could potentially lead to biased estimates of index test accuracy. Many papers in the literature discussed missing reference test outcome (missing disease status) and how to correct such bias, so called partial verification bias or work up bias [1–4]. However, index test outcomes can be non-evaluable as well, especially for tests yielding dichotomous results. Different situations were discussed where index test result can be non-evaluable: uninterpretable, intermediate and indeterminate [5, 6].

For a single study, there are many discussions about how to deal with non-evaluable index test outcomes, such as excluding them [7], grouping them with positive or negative outcomes [5, 7], or use 3×2 table to report them as an extension of the standard 2×2 table [7]. On the other hand, in meta-analysis, there is little discussion on how to deal with missing index test outcomes [6]. The “classic” 2×2 table models such as the bivariate linear mixed models [8–13], bivariate generalized linear mixed model (GLMM) [14–16] and TGLMM [17] ignore missing index test outcomes. Recently, a paper by Schuetz et al. [6] discussed this issue by studying different approaches dealing with index test non-evaluable subjects. The paper conducted a meta-analysis of coronary CT angiography studies and presented an intent-to-diagnose approach together with three commonly applied alternative approaches. The intent-to-diagnose approach takes non-evaluable diseased subjects as false positives and non-diseased subjects as false negatives such that sensitivity and specificity won’t be over-estimated. We name the other three alternative approaches in Schuetz et al. [6] as Model 1 (non-evaluable subjects are excluded from the study), Model 2 (non-evaluable diseased subjects are taken as true positives and non-diseased subjects are taken as false positives) and Model 3 (non-evaluable diseased subjects are taken as false negatives and non-diseased subjects are taken as true negatives). We use Model 1-3 to denote the above three approaches thoughout the rest of this paper. The authors concluded that excluding the index test non-evaluable subjects (Model 1) leads to overestimation of sensitivity and specificity and recommended the conservative intent-to-diagnose approach by treating non-evaluable diseased subjects as false negatives and non-evaluable non-diseased subjects as false positives. However, no simulation studies have been conducted to evaluate the performance of these approaches. Moreover, the above conclusions can be misleading.

We can treat index test non-evaluable subjects as missing data. Schuetz et al. [6] concluded that sensitivity and specificity could be over-estimated by excluding non-evaluable subjects. In fact, under a reasonable general assumption, missing at random (MAR), excluding non-evaluable subjects can provide unbiased estimates of sensitivity (Se) and specificity (Sp). Under MAR assumption, the probability of missing only depend on observed information, such as patient characteristics and known true disease status [18, 19]. For example, when diagnosing extrahepatic cholestasis using percutaneous transhepatic cholangiography, non-diseased subjects can have uninterpretable results more often than diseased patients [5]. A special case of MAR is missing completely at random (MCAR), where missing is independent of both observed and unobserved variables [18]. E.g., accidental contamination of a urine sample such that the test result is discarded. Under MAR, *T* and *M* are independent given disease status *D*, where *M*=1,0 indicates missingness of index test outcome, *D*=1,0 indicates diseased or non-diseased and *T*=1,0 represents index test positive or negative. Hence, excluding non-evaluable subjects will have unbiased estimates of Se and Sp:  and . Similarly, positive and negative likelihood ratios (LR + and LR −) and area under the curve (AUC) are unbiased too. Under MCAR, *Pr*(*M*=1|*D*=1)=*Pr*(*M*=1|*D*=0), and hence disease prevalence (*π*) estimate is also unbiased if non-evaluable subjects are excluded. However, when missing probabilities are not equal between diseased and non-diseased participants, disease prevalence estimate can be biased if non-evaluable subjects are excluded, leading overall estimates of positive predictive value (PPV) and negative predictive value (NPV) biased. PPV and NPV are generally preferred by clinicians as measurements of how well a test predicts true disease status because their interpretations are more intuitive: PPV is the probability that a subject with positive intex test result is truely diseased and NPV is the probability that a subject with negative intex test result is truely non-diseased [19]. However, none of the approaches discussed in Schuetz et al. [6] can correct bias in their estimates.

In this article, we propose to extend the TGLMM approach [17] by treating non-evaluable subjects as missing data to adjust for potential bias. The TGLMM was proposed by Chu et al. [17] as an extension of the bivariate GLMM [9, 10, 14]. Sensitivities and specificities are found to be potentially dependent on disease prevalence [20–22]. The TGLMM models disease prevalence together with sensitivity and specificity to account for potential correlations among them. Moreover, once overall disease prevalence is evaluated, other test accuracy indices such as PPV and NPV can be calculated. By extending the TGLMM to account for missing data, potential bias in disease prevalence estimate can be adjusted and thus, bias in PPV and NPV estimates can be avoided.

In the rest of this paper, we first present the extended TGLMM approach in the “Methods” section. Next, in section “Results”, simulation studies are carried out to systematically evaluate the performance of the extended TGLMM, Model 1-3 and the intent-to-diagnose approach when there are non-evaluable index test subjects. The meta-analysis of coronary CT angiography studies is re-evaluated by the extended TGLMM approach. The SAS code for the extended TGLMM is available in the Appendix: SAS code of the extended TGLMM approach: meta-analysis of coronary CT angiography studies. Finally, we conclude the paper with some discussions in section “Conclusions”.

## Methods

Assume there are *i*=1,…,*N* studies in one meta-analysis data set. We generalize the TGLMM approach to account for missing index test outcomes by extending the “classic” 2×2 table to Table 1. Each cell in Table 1 reports the cell count and cell probability corresponding to a combination of index test and disease outcomes in study *i*. Let *n*_*itd*_ denote the cell counts in study *i* with index test outcome *T*=*t* and reference test outcome *D*=*d*, where *t*=1,0,*m* stands for positive, negative and missing, and *d*=1,0 denotes positive and negative. *Se*_*i*_, *Sp*_*i*_ and *π*_*i*_ are sensitivity, specificity and prevalence of study *i*, respectively. Let *ω*_*imd*_ denote the missing probability of index test given disease status *d* in study *i*: *ω*_*imd*_=*Pr*(*T*=*m*|*D*=*d*). The missing probabilities and disease prevalence are incorporated in the cell probabilities in Table 1. Assuming a multinomial distribution, the likelihood for ***θ***_*i*_=(*Se*_*i*_,*Sp*_*i*_,*π*_*i*_) and ***ω***_*i*_=(*ω*_*im*1_,*ω*_*im*0_) given data (cell counts) is:
1

**Table 1:** **3 × 2 table accounting for prevalence and missing index test results**

	Gold standard		
Index test	***+***	***−***	Total
	*n*_*i*11_	*n*_*i*10_	*n*_*i*1+_
+	(1−*ω*_*im*1_)*π*_*i*_ *S* *e*_*i*_	(1−*ω*_*im*0_)(1−*π*_*i*_)(1−*S* *p*_*i*_)	(1−*ω*_*im*1_)*π*_*i*_ *S* *e*_*i*_+(1−*ω*_*im*0_)(1−*π*_*i*_)(1−*S* *p*_*i*_)
	*n*_*i*01_	*n*_*i*00_	*n*_*i*0+_
−	(1−*ω*_*im*1_)*π*_*i*_(1−*S* *e*_*i*_)	(1−*ω*_*im*0_)(1−*π*_*i*_)*S* *p*_*i*_	(1−*ω*_*im*1_)*π*_*i*_(1−*S* *e*_*i*_)+(1−*ω*_*im*0_)(1−*π*_*i*_)*S* *p*_*i*_
	*n*_*im*1_	*n*_*im*0_	*n*_*im*+_
Missing	*ω*_*im*1_ *π*_*i*_	*ω*_*im*0_(1−*π*_*i*_)	*ω*_*im*1_ *π*_*i*_+*ω*_*im*0_(1−*π*_*i*_)
	*n*_*i*+1_	*n*_*i*+0_	*n*_*i*++_
Total	*π*_*i*_	1−*π*_*i*_	1

Each cell reports the cell count and cell probability corresponding to a combination of index test and disease outcomes in study *i*. *n*_*itd*_ denotes the cell counts in study *i* with index test outcome *T* =*t* and reference test outcome *D* =*d*, where *t* = 1,0,*m* stands for positive, negative and missing, and *d* = 1,0 denotes positive and negative. *Se*_*i*_, *Sp*_*i*_ and *π*_*i*_ are sensitivity, specificity and prevalence of study *i*, respectively. *ω*_*imd*_ denotes the missing probability of index test given disease status *d* in study *i*.

It is straight forward to tell from (1) that *L*(***θ***_*i*_,***ω***_*i*_|Data)∝*L*(***θ***_*i*_|Data)×*L*(***ω***_*i*_|Data), where the log-likelihood of ***θ***_*i*_ is:


Let ***θ***={***θ***_*i*_}. Assuming independence among studies conditional on ***θ***_*i*_, the total log likelihood of ***θ*** is:
2

Let logit(*π*_*i*_)=*η*+*ε*_*i*_, logit(*Se*_*i*_)=*α*+*μ*_*i*_ and logit(*Sp*_*i*_)=*β*+*ν*_*i*_, where logit(·) is the logit link function such that logit(*p*)=log(*p*/(1−*p*)), for 0<*p*<1. (*η*,*α*,*β*) are the fixed effect parameters such that median *π*, *Se* and *Sp* can be approximated as logit^−1^(*η*), logit^−1^(*α*) and logit^−1^(*β*), respectively, where logit^−1^(·) is the inverse logit function such that logit^−1^(*x*)=1/(1+exp(−*x*)). The random effect vector (*ε*_*i*_,*μ*_*i*_,*ν*_*i*_) is assumed to be trivariate normally distributed:


where the diagonal elements in ***Σ*** account for between-study variations of *π*, *Se* and *Sp* and the off-diagnonal elements take care of potential correlations among the three parameters.

Median PPV, NPV, LR + and LR − and median area under the curve (AUC_*M*_) can be approximated as [16]:


The extended TGLMM can be fitted by standard software like SAS NLMIXED procedure, which implements an adaptive Gaussian quadrature to approximate the log-likelihood in (2) integrated on random effects with dual quasi-Newton optimization techniques. The NLMIXED procedure directly outputs fixed effects estimates ,  and  and can provide median prevalence, Se, Sp, PPV, NPV, LR +, LR − estimates and their confidence intervals through the “estimate” statements. Sample SAS code is available in the Appendix: SAS code of the extended TGLMM approach: meta-analysis of coronary CT angiography studies.

## Results

### Simulations

#### Simulation scenarios

We conduct simulation studies under three missing scenarios to systematically evaluate the performance of the proposed extended TGLMM approach and the approaches discussed in Schuetz et al. [6]: missing probabilities for diseased and non-diseased subjects are same (0.1), or missing probability of diseased group (0.1) is smaller than non-diseased group (0.2), or missing probability of diseased group (0.2) is larger than non-diseased group (0.1). All three scenarios satisfy the MAR assumption, and the first scenario is in fact MCAR [18]. True sensitivity and specificity are 0.7 and 0.9, disease prevalence is 0.25 and variances of Se, Sp and prevalence are 1 on logit scale. These assumptions mimic a diagnostic test with relatively low sensitivity, high specificity and a disease with moderate prevalence. A moderate positive correlation of 0.3 is assumed between Se and *π*, and moderate negative correlations of −0.3 are assumed between Sp and *π* and between Se and Sp, on logit scales. Such correlation directions were observed in some meta-analysis studies [11, 20]. Intuitively, a population with higher prevalence may have more diseased cases with clear disease symptoms, leading to increased sensitivity. Under each setting, 5000 meta-analysis data sets are simulated with 30 studies in each data set. *π*_*i*_,*Se*_*i*_ and *Sp*_*i*_ for each study were generated according to the trivariate assumption described in the Methods section. True and false positives, true and false negatives and non-evaluable counts are sampled from the multinomial distribution in Table 1. For each simulated meta-analysis data set, the extended TGLMM, Model 1-3 and the intent-to-diagnose approach are fitted. Bias in percentage, mean standard error (SE) and 95% confidence interval coverage probability (CP) are collected and compared for estimates of sensitivity, specificity, prevalence, PPV, NPV, LR + and LR −. Bias in percentage is calculated by , where *δ* is the true value and  is the estimator.

#### Simulation results

Table 2 shows the simulation results under different scenarios. When MCAR (*ω*_*m*1_=*ω*_*m*0_=0.1), disease prevalence estimates from all five models are nearly unbiased (bias less than 1%). The extended TGLMM and Model 1 both give nearly unbiased estimates (bias less than 1.6%) and nominal coverage probabilities around 93% for Se, Sp, PPV, NPV, LR+ and LR − estimates. Model 2 over-estimates sensitivity and under-estimates specificity: bias of sensitivity estimate is 4.6% and bias of specificity estimates is 11.9%. Estimates of PPV and LR+ are more biased (22.6% bias for PPV and 49.2% bias for LR+). Using Model 3 sensitivities are largely under-estimated (12.6% bias) and specificities are over-estimated (1.1% bias). The intent-to-diagnose approach largely under-estimates both sensitivity and specificity (12.6% and 11.9% bias, respectively). The CPs for some estimates from Model 2 and 3 and the intent-to-diagnose approach can be as low as 0 (e.g., specificity estimates from Model 2), indicating that none of the confidence intervals cover the true values. When missing probability of the diseased group is smaller than the non-diseased group (*ω*_*m*1_=0.1,*ω*_*m*0_=0.2), the extended TGLMM and Model 1 both give nearly unbiased estimates (bias around 0.1%) of sensitivity and specificity. However, Model 1 over-estimates disease prevalence (9.6% bias) while the extended TGLMM gives nearly unbiased (bias within 1%) estimate of prevalence. As a consequence, Model 1 gives biased estimates of PPV and NPV (3.1% and 1.3%, respectively), while the extended TGLMM provides nearly unbiased estimates for all parameters (within 2%). Again, under this scenario, the intent-to-diagnose approach largely under-estimates sensitivity, specificity, PPV, NPV and LR+ and over-estimates LR −, with CPs less than 40% and some as low as 0. On the other hand, when *ω*_*m*1_=0.2 and *ω*_*m*0_=0.1, the extended TGLMM and Model 1 again give nearly unbiased estimates (bias around 0.1%) of sensitivity and specificity. Model 1 under-estimates disease prevalence (8.4% bias) while the extended TGLMM provides nearly unbiased estimates. The intent-to-diagnose approach largely under-estimates sensitivity, specificity, PPV, NPV and LR+ and over-estimates LR − and some CPs are as low as 0. When the missing probabilities for diseased and non-diseased subjects are more unbalanced, we expect the estimates from Model 1-3 and the intent-to-diagnose approach to have larger bias and smaller CP. In practice, however, depending on the test performance and missing probabilities, the direction and magnitude of the bias from the four approaches discussed in Schuetz et al. [6] can be different from what we observed in these simulation studies.

**Table 2:** **Simulation results under MAR assumption**

Model	TGLMM			Model 1			Model2			Model3			Intent-to-diagnose		
Estimate	Bias%	meanSE	CP	Bias%	meanSE	CP	Bias%	meanSE	CP	Bias%	meanSE	CP	Bias%	meanSE	CP
***ω***_***m1***_ ***=ω***_***m0***_ ***=0.1***															
Se	−0.3	0.041	0.94	−0.3	0.041	0.94	4.6	0.036	0.81	−12.6	0.037	0.33	−12.6	0.036	0.33
Sp	−0.1	0.017	0.93	−0.1	0.017	0.93	−11.9	0.018	0	1.1	0.015	0.84	−11.9	0.017	0
Prev	0.8	0.034	0.93	0.8	0.034	0.93	0.8	0.034	0.93	0.8	0.034	0.93	0.8	0.034	0.93
PPV	−0.1	0.046	0.94	−0.3	0.046	0.94	−22.6	0.047	0.08	−0.9	0.046	0.94	−29	0.049	0.01
NPV	−0.1	0.018	0.93	−0.1	0.018	0.93	−0.2	0.018	0.93	−2.9	0.020	0.81	−4.6	0.022	0.59
LR+	1.6	1.188	0.92	1.6	1.189	0.93	−49.2	0.307	0	−0.5	1.160	0.92	−57.6	0.271	0
LR −	0.9	0.044	0.94	0.9	0.044	0.94	1.5	0.044	0.94	27.9	0.039	0.33	46.8	0.045	0.04
***ω***_***m1***_ ***=0.1,ω***_***m0***_ ***=0.2***															
Se	−0.1	0.041	0.94	−0.1	0.041	0.94	4.7	0.036	0.80	−12.3	0.036	0.34	−12.3 0.036	0.34	
Sp	−0.1	0.017	0.94	−0.1	0.017	0.94	−22.3	0.017	0	2.2	0.014	0.62	−22.3	0.017	0
Prev	0.4	0.034	0.93	9.6	0.036	0.90	0.4	0.034	0.93	0.4	0.034	0.93	0.4	0.034	0.93
PPV	−0.3	0.046	0.93	3.1	0.044	0.88	−36	0.047	0	2.7	0.044	0.89	−42.1	0.047	0
NPV	−0.1	0.018	0.94	−1.3	0.020	0.93	−1.4	0.020	0.92	−2.7	0.020	0.83	−6.3	0.024	0.36
LR+	1.4	1.195	0.94	1.4	1.194	0.94	−65.1	0.159	0	12.3	1.312	0.95	−70.8	0.147	0
LR −	0.6	0.044	0.93	0.6	0.044	0.93	14.7	0.050	0.85	26.1	0.038	0.39	66.1	0.051	0
***ω***_***m1***_ ***=0.2,ω***_***m0***_ ***=0.1***															
Se	-0.1	0.023	0.93	-0.1	0.023	0.93	8.7	0.018	0.12	-21	0.020	0	-21	0.019	0
Sp	0	0.009	0.93	0	0.009	0.93	-10.6	0.009	0	1.1	0.008	0.74	-10.6	0.009	0
Prev	0	0.018	0.93	-8.4	0.017	0.72	0	0.017	0.91	0	0.017	0.91	0	0.0168	0.89
PPV	-0.1	0.025	0.93	-3.7	0.027	0.83	-19.1	0.025	0	-4	0.026	0.8	-30.6	0.025	0
NPV	0	0.010	0.92	1.1	0.009	0.76	1.1	0.009	0.74	-4.6	0.011	0.05	-6.2	0.012	0
LR+	0.3	0.655	0.93	0.3	0.653	0.93	-44.1	0.196	0	-11.7	0.570	0.62	-59.3	0.154	0
LR −	0.3	0.025	0.93	0.3	0.025	0.93	-10.8	0.022	0.62	47.4	0.021	0	66.7	0.024	0

Three scenarios are studied: equal or unequal missing probabilities for the diseased and non-diseased groups. Bias in percentage(Bias%), mean standard error (meanSE) and 95% confidence interval coverage probability (CP) are summarized for estimates of sensitivity (Se), specificity (Sp), prevalence (Prev), positive predictive value (PPV), negative predictive value (NPV), positive likelihood ratio (LR+) and negative likelihood ratio (LR −) from different models. “TGLMM” stands for the extended TGLMM. Model 1 excludes non-evaluable subjects, Model 2 takes non-evaluable subjects as index test positives, Model 3 takes non-evaluable subjects as index test negatives and the intent-to-diagnose approach takes non-evaluable subjects as false positives and false negatives.

### Re-evaluation of the meta-analysis of coronary CT angiography studies

Cardiac CT scans can be used to rule out stenoses, however, are found to be subject to non-evaluable results. Schuetz et al. [6] performed a systematic search for diagnostic accuracy studies of coronary CT angiography. The authors searched Medline, Embase and ISI Web of Science databases for prospective studies using conventional coronary angiography as the gold standard and have patients with non-evaluable CT images. Eventually, 26 studies were included that reports cell counts in a 3×2 table as Table 1. The authors mentioned that the 3×2 table can be extended to a 3×3 table for non-evaluable results of the gold standard, however such cases were rare (0.1%) in this systematic review. We re-evaluate the 26 studies by the extended TGLMM and compare to the estimates following the four approaches discussed in Schuetz et al. [6].

**Figure 1:**
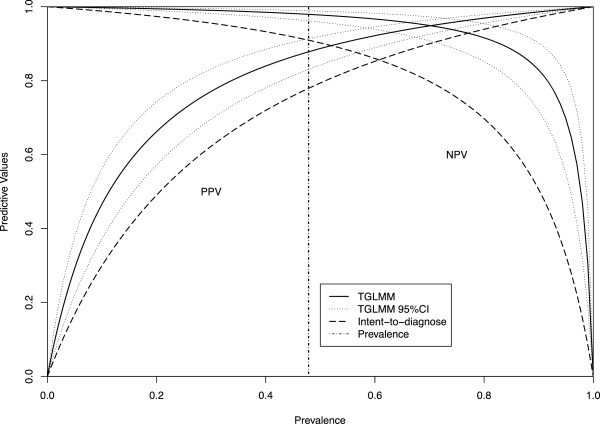
**Overall PPV and NPV plot based on the extended TGLMM (denoted by “TGLMM”) and the intent-to-diagnose approach.** The solid and dashed lines are the overall estimates of PPV and NPV from the extended TGLMM and the intent-to-diagnose approach corresponding to different prevalences ranging from 0 to 1, respectively. The dotted lines are the 95% confidence intervals of PPV and NPV estimates from the extended TGLMM approach. The vertical dashed line is the overall prevalence estimates from the meta-analysis of coronary CT angiography studies.

The fitted median estimates and 95% confidence intervals are reported in Table 3. The extended TGLMM accounting for missing subjects gives median sensitivity, specificity, LR+, LR − and AUC estimates close to the estimates when non-evaluable subjects are excluded as in Model 1. The median disease prevalence estimated from the extended TGLMM is slightly lower than the estimate from Model 1. Model 2 gives significantly lower specificity estimate and Model 3 gives lower sensitivity estimate. The intent-to-diagnose approach provides lower estimates for sensitivity, specificity and AUC as it is the most conservative approach. Figure 1 presents the estimated PPV and NPV with 95% confidence bands versus prevalence, based on the overall sensitivity and specificity estimates from the extended TGLMM and the intent-to-diagnose approach. Figure 1 shows that as disease prevalence changes, PPV and NPV estimates from the latter approach are not ever included in the 95% confidence band of the estimates from the extended TGLMM, which suggests potential underestimation of PPV and NPV.

**Table 3:** **Median estimates and 95% CI (in brackets) for parameter estimates using different methods**

Method		Sensitivity		Specificity		Prevalence		PPV
TGLMM		98.0 (96.7,99.3)		87.5 (82.7,92.3)		47.8 (37.9,57.7)		87.8 (83.3,92.3)
Model 1		98.0 (96.7,99.3)		87.4 (82.5, 92.3)		49.3 (38.9,59.7)		88.4 (84,92.7)
Model 2		98.1 (96.9,99.3)		75.9 (69.3,82.5)		47.8 (37.9,57.8)		78.9 (71.9,85.9)
Model 3		91.7 (88.1,95.4)		89 (85.4,92.7)		47.8 (37.9,57.7)		88.4 (84.1,92.7)
Intent-to-diagnose		91.7 (88.1,95.3)		76.2 (69.7,82.6)		47.9 (37.9,57.9)		78 (70.2,85.7)
**Method**		**NPV**		**LR+**		**LR −**		**AUC**
TGLMM		97.9 (96.4,99.5)		7.8 (4.8,10.9)		0.02 (0.01,0.04)		0.99 (0.96,1)
Model 1		97.8 (96.1,99.4)		7.8 (4.8,10.9)		0.02 (0.01,0.04)		0.99 (0.96,1)
Model 2		97.8 (96.2, 99.4)		4.1 (2.9,5.2)		0.02 (0.01,0.04)		0.98 (0.97,1)
Model 3		92.1 (88.4,95.8)		8.4 (5.5,11.3)		0.09 (0.05,0.14)		0.96 (0.93,0.99)
Intent-to-diagnose		90.9 (86.4,95.5)		3.8 (2.7,5.0)		0.11 (0.06,0.16)		0.93 (0.89,0.96)

“TGLMM” stands for the extended TGLMM. Model 1 excludes non-evaluable subjects, Model 2 takes non-evaluable subjects as index test positives, Model 3 takes non-evaluable subjects as index test negatives and the intent-to-diagnose approach takes non-evaluable subjects as fasle positives and false negatives. Positive predictive value (PPV), negative predictive value (NPV), positive likelihood ratio (LR+), negative likelihood ratio (LR −) and area under the curve (AUC) are summerized.

## Discussions

Adequate reporting of the missing outcomes in study reports is essential to apply the discussed models. As shown in the simulation studies, different missing scenarios can have different impact on how estimates are biased and more importantly, missing mechanism can indicate whether the MAR assumption holds. When the MAR assumption is violated, i.e., the probability of non-evaluation depends on unobserved index test outcomes, the direction and magnitude of bias are hard to predict. Few sensitivity analysis methods using pattern mixture models and selection models are available for this scenario [23, 24]. These approaches can be explored in further research. On the other hand, number of non-evaluable results need to be known in order to apply the proposed methods. However, a recent study shows that they are not consistently or adequately reported in published studies [25].

A reviewer has pointed out that as long as number of non-evaluable subjects are known, disease prevalence can be estimated unbiasedly through an univariate meta-analysis. Consequently, together with unbiased sensitivity and specificity estimates, PPV and NPV estimates are unbiased too. This approach is a simpler method than the proposed extended TGLMM to estimate prevalence, however, can be less efficient by ignoring the potential correlation between prevalence, sensitivity and specificity, which may result in wider confidence intervals.

For an individual patient, different approaches of treating a missing result can have different impact. For example, if index test results are missing due to the same reason of returning a negative result (and thus is MNAR), then treating such patients as disease negatives can yield unbiased estimate of prevalence for a study, and also won’t affect the patients’ diagnosis. On the contrary, if index test missing patients are treated as positives for reasons such as suspicious of serious disease like cancer [26], it may result in over-estimation of disease prevalence and unnecessary medial cost for the patient. For another example, if index test is repeatable and repeated for subjects with non-evaluable results, then it is appropriate to ignore missing results.

## Conclusions

In this paper, we propose an extended TGLMM approach to handle non-evaluable index test subjects in meta-analysis of diagnostic tests. The extended TGLMM is compared to an intent-to-diagnose approach and three alternative approaches proposed by Schuetz et al. [6] through simulation studies and re-evaluaion of the meta-analysis of coronary CT angiography studies.

In summary, by simulation studies we showed that under MAR assumption, excluding index test non-evaluable subjects (Model 1) will not lead to biased estimates of sensitivity, specificity, LR+, LR − and AUC. Thus in practice, researchers can be confident to apply Model 1 when there is a belief in the MAR assumption. However, when disease prevalence or PPV and NPV are of interest, excluding non-evaluable subjects could lead to biased estimates of these parameters. Under this situation, the extended TGLMM accounting for missingness should be preferred. Even though the extended TGLMM is more theoretically complex than the widely used bivariate random effects model, it is easy to program use SAS NLMIXED procedure. Sample SAS code with an application to the meta-analysis of coronary CT angiography studies is provided in the Appendix: SAS code of the extended TGLMM approach: meta-analysis of coronary CT angiography studies. Model 2, Model 3 and the intent-to-diagnose approach all largely under- or over- estimate sensitivity and specificity, so that they should not be recommended when MAR assumption is not seriously violated.

## Claims

Ethical approvals and informed consents are not applicable to this paper.

## Appendix: SAS code of the extended TGLMM approach: meta-analysis of coronary CT angiography studies


